# Monotherapy for low-risk gestational trophoblastic neoplasia with score 5-6

**DOI:** 10.3389/fonc.2022.1035170

**Published:** 2022-11-09

**Authors:** Li Kemin, Zhang Mengpei, Yin Rutie

**Affiliations:** ^1^ The Department of Obstetrics and Gynecology, West China Second University Hospital of Sichuan University, Chengdu, Sichuan, China; ^2^ Key Laboratory of Birth Defects and Related Diseases of Women and Children, Sichuan University, Ministry of Education, Chengdu, Sichuan, China

**Keywords:** monotherapy for GTN with score 5-6 monotherapy showed remarkable advantages low-risk, gestational trophoblastic neoplasia (GTN), single-agent chemotherapy, combination chemotherapy, FIGO/WHO prognostic score

## Abstract

**Objective:**

To investigate the monotherapy for gestational trophoblastic neoplasia (GTN) patients with FIGO/WHO prognostic score of 5–6.

**Methods:**

The low-risk GTN patients from 2012 to 2019 were enrolled. The study is a retrospective report to analyze the efficacy and safety of single-agent chemotherapy and combination chemotherapy in patients with a high FIGO/WHO prognostic score of 5–6.

**Results:**

75 cases (33.5%) were included. Complete remission was in all patients. Among the 29 cases taking single-agent chemotherapy, 22 cases (75.9%) developed drug resistance. Among the 46 cases taking combination chemotherapy, 7 patients (15.2%) developed drug resistance. There was a statistically significant difference in the drug resistance rate between these two subgroups (P < 0.05), but there was not statistically significant difference in the total number of chemotherapy courses (<2mIU/ml) (P < 0.05).

**Conclusion:**

Monotherapy showed remarkable advantages in GTN patients with FIGO/WHO prognostic score of 5–6.

## Highlights:

• Monotherapy for GTN with score 5-6

• Monotherapy showed remarkable advantages

## Introduction

Gestational trophoblastic neoplasia (GTN) is the only gynecological malignant tumor related to pregnancy that can be cured by chemotherapy (1, 2). The FIGO (International Federation of Gynecology and Obstetrics)/WHO (World Health Organization) prognostic scoring system (2000) divides GTN into low-risk (≤6 points) and high-risk (≥7 points) types and suggests stratified treatment (World Health Organization scoring system is based on prognostic factors, including age, antecedent pregnancy, Interval from index pregnancy, months, Pretreatment hCG, Largest tumor size, Site of metastases, Number of metastases identified, Previous failed chemotherapy. See FIGO guidelines (1)). The 2018 FIGO guidelines (1) and the 2021 NCCN (National Comprehensive Cancer Network) Clinical Practice Guidelines in Oncology (2) both recommend single-agent chemotherapy as a first-line regimen for low-risk patients using actinomycin-D (KSM) or methotrexate (MTX). Some scholars believe that the efficacy of KSM is significantly better than that of MTX (3).

Epidemiological surveys show that the incidence of mole in China and some parts of Asia is 2/1,000 pregnancies (4). The incidence of choriocarcinoma is low. Due to the lack of histopathological evidence in many clinical cases, it is difficult to distinguish choriocarcinoma after hydatidiform mole from invasive hydatidiform mole, so the exact incidence is difficult to estimate, which is 1/40 000~9/40 000 pregnancies (4). The treatment standards in China are different slightly from NCCN guideline or FIGO guideline. For low-risk patients, single-agent chemotherapy can be used. For low-risk patients with a prognostic score of 5-6 or a pathological diagnosis of choriocarcinoma, the risk of failure of first-line single-agent chemotherapy is significantly increased, and combined chemotherapy can be selected according to the regimen of patients with a high prognostic score (4).

Patients with metastatic disease, FIGO scores of 5-6, or histologic diagnosis of choriocarcinoma are more likely to require second-line therapy than patients with nonmetastatic disease, FIGO risk scores lower than 4, and patients who do not have choriocarcinoma (1, 5). Overall, 85–95% of low-risk patients can be cured without multiagent chemotherapy or hysterectomy. The overall cure rate for patients with low-risk disease approaches 100% (1, 2, 5).

Several controversies exist within the risk score with one of the current key challenges being whether low-risk patients scoring 5-6 should be still considered low risk and initially treated with single-agent chemotherapy, given that these patients have a higher risk of resistance to single-agent chemotherapy (6).

Although this disease is extremely sensitive to chemotherapy, many patients still develop first-line single-agent drug resistance. Studies have shown that in low-risk patients, the probability of changing from single-agent to combination chemotherapy due to drug resistance is 9–33% (7, 8). The rate of resistance to first-line single-agent chemotherapy in low-risk GTN patients with a high FIGO/WHO prognostic score of 5–6 has been shown to be 14 times higher than that in patients with a low FIGO/WHO prognostic score of 0–4 (9). It is still controversial whether combination chemotherapy regimens work better. The West China Second University Hospital of Sichuan University is the largest gynecological cancer center in western China, and we have treated a total of 627 inpatients with GTN in the past eight years, among which either patients with FIGO/WHO prognostic scores of 5–6 or patients with lung metastasis were more than 30%. In the present study, the low-risk GTN inpatients in the West China Second Hospital of Sichuan University from 2012 to 2019 were taken as the research object to explore the chemotherapy regimen options for low-risk GTN patients with high FIGO/WHO prognostic score 5–6, serving as a basis for the clinical treatment.

## Materials and methods

### Research object

The study is a retrospective report. The patients with low-risk GTN who were admitted to the West China Second University Hospital of Sichuan University from 2012 to 2019 were the subjects of the present study. We collected clinical data of study subjects from electronic medical records system. The inclusion criteria were: (1) Stage I–III according to the 2000 FIGO GTN anatomical staging, with a FIGO/WHO prognostic score 5-6; (2) comprehensive medical history and physical examination conducted before chemotherapy, in addition to complete auxiliary examination data including routine blood work, liver and kidney function, human chorionic gonadotropin (β-hCG) level, abdominal and pelvic ultrasound, lung plain films (for quantitation of the number of metastatic lesions), and computerized tomography scans (CT, for screening of metastatic lesions of all organs), magnetic resonance imaging (MRI, for patients with suspected brain metastases); (3)chemotherapy regimen in accordance with the NCCN or FIGO guidelines during treatment; and (4) regular follow-up. The exclusion criteria were: (1) confirmed pathology of placental site trophoblastic tumor (PSTT), choriocarcinoma or epithelioid trophoblastic tumor (ETT); (2) receipt of non-standard treatment; (3) receipt of germicidal treatment such as MTX or traditional Chinese medicine; and (4) coexistence of other malignant tumors.

### Study groups and treatment plan

Patients were divided into a single-agent chemotherapy group and a combination chemotherapy group according to their regimen at the time of admission. The single-agent regimens include 5-day Methotrexate (MTX) or Actinomycin D, and the combination regimens include Methotrexate (MTX) +Actinomycin D or EMA-CO.

### Efficacy and observation indexes

Efficacy index: The serum β-hCG level was rechecked before each course of chemotherapy for efficacy evaluation. When the β-hCG level dropped below 2mIU/ml, it was considered a serological complete response (CR), and 2–3 more courses of consolidation chemotherapy were given in addition to follow-up. If the β-hCG level dropped less than one logarithmic scale or even increased for two consecutive tests, or the imaging examination indicated no shrinkage or even increased size or new lesions, it was considered ineffective or drug-resistant, and the chemotherapy regimen needed to be changed and a new efficacy evaluation performed. One month after chemotherapy was stopped, if the CR patient had a β-hCG level above the normal range for two consecutive tests, it was considered recurrence (after excluding pregnancy).

The drug resistance rate was defined as the proportion of patients who changed chemotherapy regimens due to drug resistance during chemotherapy. In single-agent chemotherapy group, if HCG dropped unsatisfactory or even raised, patients would be undergone another single-agent chemotherapy. HCG dropped unsatisfactory or even raised again, patients were changed to the combination chemotherapy group. It is defined as drug resistance. However, HCG dropped unsatisfactory or even raised in combination chemotherapy group, patients would be undergone another combination chemotherapy. It is also defined as drug resistance.

Observation indexes: The drug resistance rate, total number of chemotherapy courses, number of chemotherapy courses required for β-hCG to return to normal (<2mIU/ml), and adverse chemotherapy reactions.

### Adverse reaction evaluation

All patients had routine blood work and liver and kidney function evaluation before chemotherapy and within one week of stopping treatment. Evaluation for adverse chemotherapy reactions was performed following the National Cancer Institute Common Toxicity Criteria 4.0 (NCI-CTC4.0) (7).

### Statistical analysis

SAS 9.2 was used for statistical analysis. The measurement data are presented as the mean ± standard deviation, and the count data are expressed as a percentage. The comparison between groups was conducted using a *t*-test, and P <0.05 was considered a statistically significant difference.

## Results

### Basic characteristics of the included study objects

**Figure 1:**
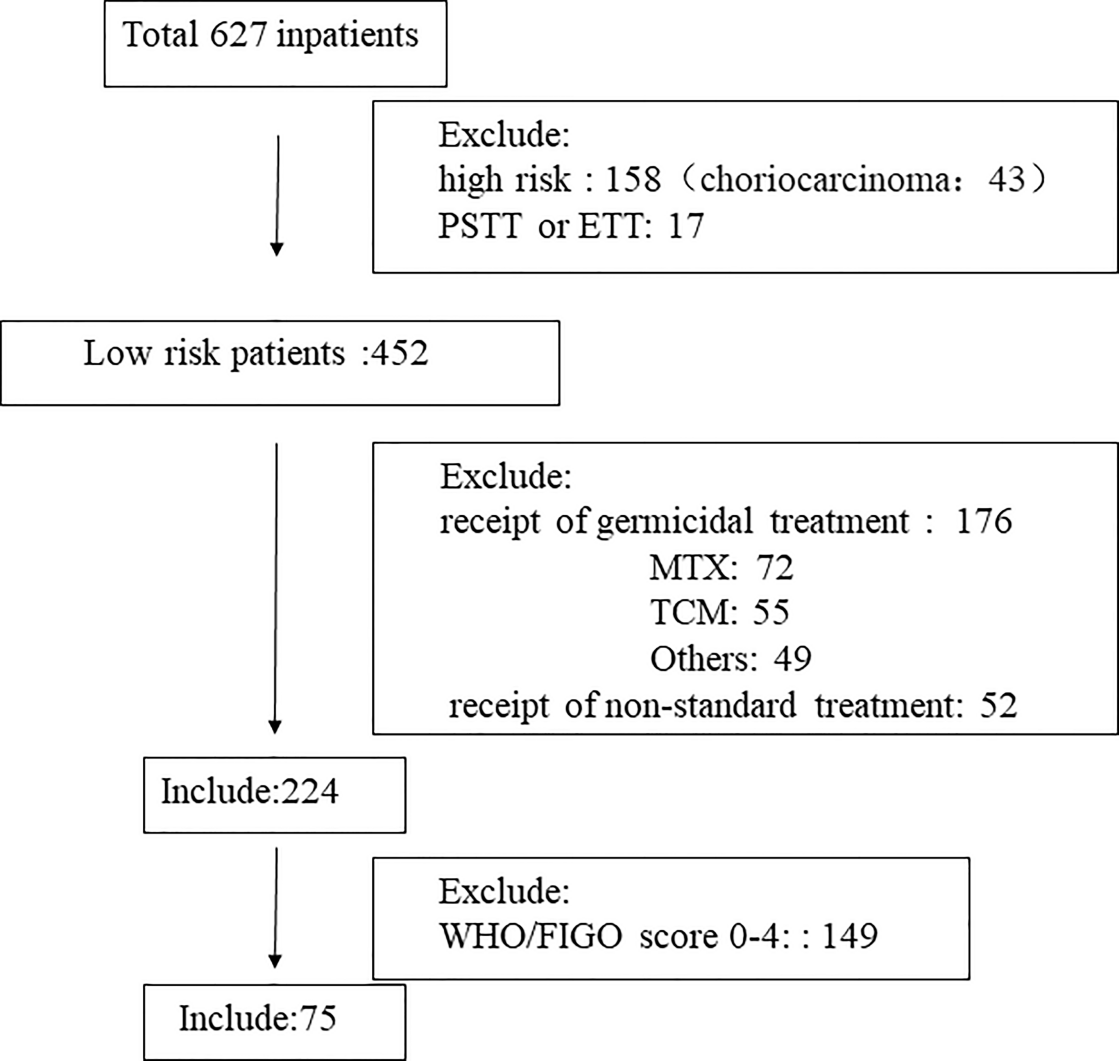
Flow diagram showing the derivation of the patient population. TCM, Traditional Chinese Medicine; MTX, methotrexate; PSTT, placental site trophoblastic tumor; ETT epithelioid trophoblastic tumor.

The basic characteristics of the included study subjects are shown in **Table 1**. A flow diagram shows the derivation of the patient population (**Figure 1**). A total of 224 low-risk GTN patients. 75 patients (33.5%) had a FIGO/WHO prognostic score of 5–6. The follow-up time was 12–74 months, during which there were 3 (1.34%) cases of recurrence and no deaths.

**Table 1:** The baseline of patients with FIGO/WHO prognostic score(5-6).

		single-agent	combination	total	P value
n		29(38.7%);	46(61.3%);	75	
age	Mean age	35.00±11.90	34.26±10.07	34.55±10.74	>0.05
	≥40 years	6 (20.7%)	9 (19.6%)	15	
FIGO stage	I-II	17 (58.6%)	28 (60.9%)	45	
	III	12 (41.4%)	18 (39.1%)	30	
pre-treatment HCG	<10^3^	3 (10.3%)	5 (10.9%)	8	
	10^3^-10^4^	12 (41.4%)	19 (41.3%)	31	
	10^4^-10^5^	9 (31.0%)	14 (30.4%)	33	
	>10^5^	5 (17.3%)	8 (17.4%)	13	
Pregnancy types	mole	20 (69.0%)	32 (69.6%)	52	
	abortion	7 (24.1%)	11 (13.9%)	18	
	term	2 (6.9%)	3 (6.5%)	5	
Interval	<4 m	15 (51.7%)	24 (52.2%)	39	
	4-6 m	8 (27.6%)	13 (28.3%)	21	
	7-12 m	6 (20.7%)	8 (17.4)	14	
	>12 m	0 (0)	1 (2.2%)	1	

FIGO, International Federation of Gynecology and Obstetrics; WHO, World Health Organization.

### Efficacy evaluation of single-agent and combination chemotherapy

The efficacy evaluation in patients with different FIGO/WHO prognostic scores is shown in **Table 2**. There were 75 patients with a FIGO/WHO prognostic score of 5–6, among which 46 (85.2%) developed drug resistance. The number of chemotherapy courses was 7.7 ± 1.8, and the number of chemotherapy courses required for β-hCG to return to normal was 5.4 ± 1.8.

**Table 2:** The efficacy evaluation of single-agent and combination chemotherapy.

	n	drug resistance rate	P value	total number of chemotherapy courses	P value	number of chemotherapy courses required for β-hCG to return to normal (<2mIU/ml)	P value
FIGO/WHO prognostic score (5-6);	75	46 (85.2%);		7.7 ± 1.8		5.4 ± 1.8	
FIGO/WHO prognostic score (5-6);and single-agent chemotherapy	29	22 (75.9%);	<0.00001	7.8 ± 2.1	0.432	5.4 ± 1.8	0.092
FIGO/WHO prognostic score (5-6);and combination chemotherapy	46	7 (15.2%);		7.4 ± 2.0		4.8 ± 1.6	
FIGO/WHO prognostic score (5-6); and FIGO III stage single-agent chemotherapy	12	11 (91.7%);	0.00002	8.4 ± 1.8	0.145	5.7 ± 1.8	0.15
FIGO/WHO prognostic score (5-6); and FIGO III stage combination chemotherapy	18	3 (16.7%);		7.5 ± 2.0		4.8 ± 1.8	

FIGO, International Federation of Gynecology and Obstetrics; WHO, World Health Organization.

There were 29 patients with a FIGO/WHO prognostic score of 5–6 who were undergoing single-agent chemotherapy, among which 22 (75.9%) developed drug resistance. The number of chemotherapy courses was 7.8 ± 2.1, and the number of chemotherapy courses required for β-hCG to return to normal was5.4 ± 1.8. There were 46 patients who were undergoing combination chemotherapy, among which 7 (15.2%) developed drug resistance. The number of chemotherapy courses was 7.4 ± 2.0, and the number of chemotherapy courses required for β-hCG to return to normal was 4.8 ± 1.6. A statistically significant difference was found in the drug resistance rate between these two subgroups (P < 0.05); however, there was no significant difference between these two subgroups in terms of the number of chemotherapy courses or the number of chemotherapy courses required for β-hCG to return to normal (P > 0.05).

### Efficacy evaluation in FIGO stage III patients

The efficacy evaluation in FIGO stage III patients is shown in **Table 2**. There were 12 FIGO stage III patients with a FIGO/WHO prognostic score of 5–6 who were undergoing single-agent chemotherapy, among which 11 (91.7%) developed drug resistance. The number of chemotherapy courses was 8.4 ± 1.8, and the number of chemotherapy courses required for β-hCG to return to normal was 5.7 ± 1.8. There were 18 FIGO stage III patients with a FIGO/WHO prognostic score of 5–6 who were undergoing combination chemotherapy, among which 3 (13.3%) developed drug resistance. The number of chemotherapy courses was 7.5 ± 2.0, and the number of chemotherapy courses required for β-hCG to return to normal was 4.8 ± 1.8. A statistically significant difference was found in the drug resistance rate between the two subgroups (P < 0.05); however, there was no significant difference between these two subgroups in terms of the number of chemotherapy courses or the number of chemotherapy courses required for β-hCG to return to normal (P > 0.05).

### Adverse reactions

The incidence of adverse reactions is shown in **Table 3**. In patients receiving combination chemotherapy, the incidence of bone marrow suppression, including decreased white blood cell count, decreased neutrophils, and anemia, was significantly higher than that in patients receiving single-agent chemotherapy (P < 0.05). The incidence of gastrointestinal adverse reactions in patients undergoing combination chemotherapy was significantly higher than that in patients undergoing single-agent chemotherapy (P < 0.05). Nevertheless, there was no significant difference in the incidence of gastrointestinal adverse reactions grade ≥3 between these two groups (P>0.05). There was no discontinuation or abandonment of treatment due to intolerable adverse reactions or any serious adverse reactions leading to death.

**Table 3:** Adverse reactions.

		single-agent chemotherapy	combination chemotherapy	Pvalue
n		12	18	
Liver damage	total	8 (66.7%)	12 (66.7%)	0.86
	≥grade 3	1 (8.3%)	0 (0)	0.42
Leucopenia	total	9 (75.0%)	17 (94.4%)	0.003
	≥grade 3	1 (8.3%)	5 (27.8%)	0.01
Neutropenia	total	9 (75.0%)	11 (61.1%)	0.006
	≥grade 3	4 (33.3%)	8 (44.4%)	0.0006
Anemia	total	8 (66.7%)	15 (83.3%)	0.007
	≥grade 3	0 (0)	2 (11.1%)	0.03
Thrombocytopenia	total	3 (25.0%)	4 (22.2%)	0.89
	≥grade 3	0 (0)	1 (5.6%)	0.34
Mouth ulcers	total	2 (16.7&)	5 (27.8%)	0.23
	≥grade 3	1 (8.3%)	0 (0)	0.2
Gastrointestinal tract	total	2 (16.7%)	11 (61.1%)	0.00001
	≥grade 3	1 (8.3%)	0 (0)	0.3
Others	total	1 (8.3%)	1 (5.6%)	0.58
	≥grade 3	0 (0)	0 (0)	–

## Discussion

FIGO adopted a prognostic score and anatomical staging system to describe the diagnosis and staging of GTN, which has been widely applied in clinical diagnosis and treatment since 2002. Prognostic scores of 0–6 and ≥7 indicate low-risk and high-risk patients, respectively (10). The existing guidelines recommend performing combination chemotherapy in high-risk patients and single-agent chemotherapy in low-risk patients based on the prognostic scoring system (1, 2).

Correctly assessing and predicting GTN chemoresistance, exploring the relevant molecular mechanisms of chemoresistance, and searching for chemoresistance predictors and potential therapeutic targets are urgent clinical needs. Previous studies have confirmed that MTX resistance is related to a variety of molecules, such as ABC transporter and multidrug resistance-related protein (MRP1) pumping out drugs, resulting in a decrease in intracellular drug concentrations (11). Dipeptidyl peptidase-4 (DPP4), methyltransferase-like protein 7A (METTL7A), and transcription factor SOX8 may promote MTX resistance by activating pro-survival signaling pathways and reducing the accumulation of reactive oxygen species (ROS) in JEG3 cells (12). Studies have found that high expression of human leukocyte antigen-G (HLA-G) is a strong candidate gene for predicting the resistance of choriocarcinoma to MTX single-agent chemotherapy (13). Although research on the mechanism of GTN resistance is still emerging, the exact mechanism of resistance remains to be further elucidated.

Studies have shown that a considerable number of patients are resistant to first-line chemotherapy agents, specifically 25–35% of patients with FIGO/WHO prognostic score ≤6 and 70–80% of patients with FIGO/WHO prognostic score 5–6 (6). The Sheffield Trophoblastic Disease Centre in the United Kingdom reported an 81% first-line single-drug resistance in patients with a FIGO/WHO score of 6, and a 34% resistance in patients with a score less than 6 (14). A recent study in Canada on low-risk GTN investigated a total of 412 patients and reported a chemotherapy failure rate of 32% for patients with a FIGO/WHO prognostic score of 0–4, and a significantly increased failure rate of 59% for patients with a FIGO/WHO prognostic score of 5–6 (15). Many scholars have debated whether the current staging and scoring system should be modified to a more precise model, so that some susceptible drug-resistant patients could adopt a more effective plan at the beginning of treatment. However, Braga A et al (16)found approximately 60% of women with gestational trophoblastic neoplasia presenting with a FIGO risk score of 5-6 could achieve remission with single-agent therapy; almost all remaining patients have complete remission with subsequent multiagent chemotherapy. Primary multiagent chemotherapy should only be given to patients with metastatic disease and choriocarcinoma, regardless of pretreatment human chorionic gonadotropin concentration, or to those defined by our new predictors including metastatic disease, choriocarcinoma and pretreatment human chorionic gonadotropin concentration. we conducted a study aimed at analyzing the effectiveness and safety of single-agent and combination chemotherapy in patients with high FIGO/WHO prognostic scores.

Our results are consistent with Braga A et al’ study (16). We excluded choriocarcinoma or FIGO stage IV patients, the drug resistance rate decreased from 75.9% for first-line single-agent chemotherapy to 15.2% for first-line combination chemotherapy, But the combination chemotherapy did not decrease the number of chemotherapy courses or the number of chemotherapy courses required for β-hCG to return to normal. And almost all remaining patients have complete remission with subsequent multiagent chemotherapy in single-agent chemotherapy group, and adverse reactions in patients undergoing combination chemotherapy regimens are significantly increased.

In recent years, immunotherapy has been more and more widely used in advanced solid tumors due to its durable efficacy and low adverse drug reactions. For low-risk GTN, about 20% to 25% of newly diagnosed patients develop resistance to single-agent methotrexate therapy, and almost all of these resistant patients can be cured by sequential single-agent or multi-agent chemotherapy with good prognosis (17). Therefore, the use of immunotherapy in such patients is currently controversial. In 2020, You et al. (18) reported a phase II clinical trial using the PD-L1 inhibitor Avelumab in patients with low-risk GTN (TROPHIMMUN cohort A), the study included 15 patients who were resistant to methotrexate or actinomycin D. The results showed that 53.3% (8 patients) achieved complete remission, and 42.3% (42.3%) of the 7 patients who were ineffective against Avelumab patients received combination chemotherapy, which is much higher than that reported in the literature for low-risk patients with methotrexate and actinomycin D resistance who required multidrug chemotherapy. Expensive treatment costs and low response rates make the application of immunotherapy in low-risk GTN patients full of controversy. Therefore, in clinical practice, salvage chemotherapy is still recommended for patients with low-risk GTN who are resistant to initial treatment with single-agent chemotherapy, and immunotherapy should be used with caution after fully weighing the pros and cons.

Although GTN distant metastases in the liver, spleen, and brain are scoring indicators in the FIGO prognostic scoring system, the guidelines state that patients with lung metastases, that is, FIGO stage III patients, have the same prognosis as stage I and II patients; Hence, lung metastasis is not used as a prognostic scoring indicator. However, the number of visible metastatic lesions in the lung is related to the prognosis of the patient. Especially for those with more than eight visible metastatic lesions in the lung, the prognosis is significantly worse; Therefore, it is recommended that GTN patients with lung metastases detected by CT or magnetic resonance imaging (MRI) should have the number of visible metastatic lesions counted by X-ray and input to the prognosis score (10). Recent studies have shown that the rate of resistance to single-agent chemotherapy in low-risk GTN patients with lung metastasis is significantly higher than that in patients without lung metastasis. A cohort study on low-risk GTN with lung metastasis included 1,040 low-risk GTN cases, 65 of which had lung metastasis and 975 did not (19). The results showed a resistance rate of 60% to initial treatment with MTX in patients with lung metastasis and 38% in low-risk patients with no lung metastasis. The FIGO/WHO score in low-risk patients with lung metastasis was significantly higher than that in low-risk patients with no lung metastasis (median prognostic score 4 (3–5) vs. 3(1–4), P <0.001). Braga A et al’ study (16) shown that metastatic disease was Predictors for single-agent resistance in FIGO score 5-6 gestational trophoblastic neoplasia, and Primary multiagent chemotherapy should be given to patients with metastatic disease. We found out that the rate of resistance to single-agent chemotherapy in low-risk GTN patients with lung metastasis was increased significantly, and that in patients with a high prognostic score (FIGO/WHO prognostic score of 5–6) and lung metastasis was as high as 92.3%. But the results in the number of chemotherapy courses or the number of chemotherapy courses required for β-hCG to return to normal or adverse reactions is the same as the nonmetastatic patients with FIGO/WHO risk score of 5-6.

However, this was a single-center, retrospective, non-randomized controlled study with a relatively small sample size. And we collected clinical data of study subjects from electronic medical records system. It is difficult to perform prospective NCI-CTC evaluation. Whether the conclusions are accurate must be confirmed by high-quality large-sample, multi-center, randomized controlled studies. A randomized controlled study of chemotherapy options for patients with a high prognostic score (FIGO/WHO prognostic score of 5–6) in China is currently in progress (NCT03885388). Our center recently joined this study as one of the research centers. We look forward to the outcomes of this study with the hope that high quality clinical research will serve as high-level evidence-based practice.

Overall, monotherapy showed significant advantages in low-risk GTN patients with a FIGO/WHO score of 5–6, with or without lung metastases.

## Data availability statement

The original contributions presented in the study are included in the article/supplementary material. Further inquiries can be directed to the corresponding author.

## Ethics statement

The studies involving human participants were reviewed and approved by Approvalled Medical Ethics Committee of West China Second University Hospital, Sichuan University. Ethical Lot Number:20200076. And exempt from signing informed consent by Medical Ethics Committee. Written informed consent for participation was not required for this study in accordance with the national legislation and the institutional requirements.

## Author contributions

Conception: LK and YR. Design: all authors. Data collection: LK and ZM. Data analysis: LK. Manuscript writing: LK and ZM. Final manuscript approval: all authors. All authors contributed to the article and approved the submitted version.

## Funding

The Key Project of Sichuan Provincial Department of Science and Technology: “Study on the key factors affecting the diagnosis and treatment of major diseases in obstetrics and gynecology (19ZDYF).

## Conflict of interest

The authors declare that the research was conducted in the absence of any commercial or financial relationships that could be construed as a potential conflict of interest.

## Publisher’s note

All claims expressed in this article are solely those of the authors and do not necessarily represent those of their affiliated organizations, or those of the publisher, the editors and the reviewers. Any product that may be evaluated in this article, or claim that may be made by its manufacturer, is not guaranteed or endorsed by the publisher.

## Abbreviations

GTN, gestational trophoblastic neoplasia; FIGO, International Federation of Gynecology and Obstetrics; WHO, World Health Organization; NCCN, National Comprehensive Cancer Network; KSM, actinomycin-D.

MTX methotrexate; β-hCG, human chorionic gonadotropin; PSTT, placental site trophoblastic tumor; ETT, epithelioid trophoblastic tumor; CR, complete response.
